# Examining the Impact of the World Health Organization 2022 Guidelines on Evaluation of Biosimilars for Non-Local Comparators in Biosimilar Studies on Middle East and North Africa Member States

**DOI:** 10.3390/pharmacy12030094

**Published:** 2024-06-16

**Authors:** Michael W. Strand, Jonathan H. Watanabe

**Affiliations:** Center for Data-Driven Drugs Research and Policy, School of Pharmacy and Pharmaceutical Sciences, University of California, 856 Health Sciences Drive Road, Irvine, CA 92697, USA; mwstrand@uci.edu

**Keywords:** biosimilars, biologics, World Health Organization, regulation, Middle East, North Africa, medicines, reference product, regulatory guidelines

## Abstract

Global support and standardization of regulation for biosimilars approval owes much of its legacy to the World Health Organization (WHO), since the first guidance by the organization on the matter was released in 2009. Since then, and with over a decade of research, the 2022 revision provides opportunities for time and financial savings to pharmaceutical manufacturers aiming to prove similarity of a potential biosimilar product to some reference product, particularly by clarifying that the use of a non-local reference product as a comparator in certain studies is permissible. This declaration has important implications, particularly in the emerging biological markets of the Middle East and North Africa region, where WHO guidelines have been integral to the regulatory framework of over a dozen countries for more than a decade. This article aims to review the impact of this revision on these countries and relevant policies on non-local comparator usage. Since 2022, this revision has been adopted only in Egypt. Many North African countries are yet to adopt a first draft of the formalized guidance. This analysis revealed that, although many of these countries reference the WHO guidelines, hesitation remains in terms of sourcing comparator products outside the US or European countries. This likely translates to slow regional development and cooperation of functioning, sustainable biosimilars markets. Future studies will be necessary to evaluate the continued development of guidance within these countries and changes in comparator sourcing norms as more time is allowed for their policies to mature and adapt to new standards.

## 1. Introduction

**Figure 1:**
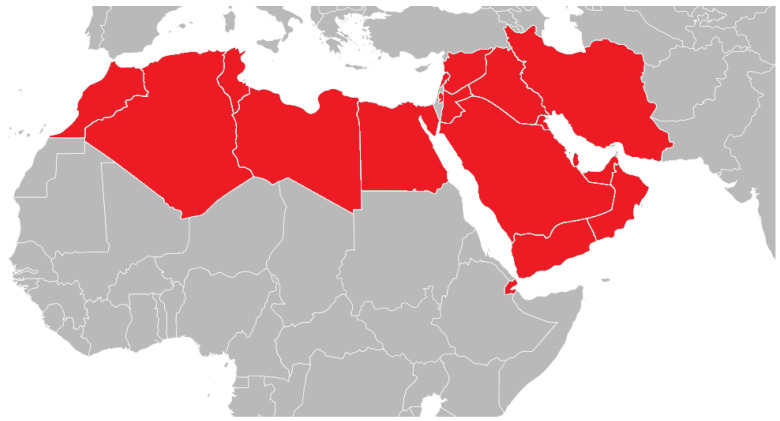
The 19 MENA countries according to the World Bank in red. From left to right: Morocco, Algeria, Tunisia, Libya, Egypt, West Bank and Gaza, Kingdom of Saudi Arabia, Jordan, Lebanon, Syria, Iraq, Djibouti, Iran, Yemen, Kuwait, Bahrain, Qatar, Oman, and United Arab Emirates.

The World Health Organization (WHO) has been instrumental in the support and development of regulation and guidance for biotherapeutic products critical to treating chronic illness and disease worldwide. Since 2009, with the adoption of the Guidelines on evaluation of Similar Biotherapeutic Products (SBPs) [1], herein referred to as the “2009 guidelines”, the WHO has worked among the 194 member states to support regulatory authorities in the creation of a standardized approach to “similar biotherapeutics”—now routinely referred to as “biosimilars”—regulation, and policy, increasing global access to these safe and effective modern medicines. The WHO has since worked continuously to improve the scope and relevance of biosimilar guidelines [2]. In the Middle East and North Africa (MENA), seen in Figure 1, many countries adopt or refer to these guidelines, in addition to those of the European Medicines Agency (EMA) and the US Food and Drug Administration (FDA), when establishing or revising regulatory frameworks.

Being safe, effective, less expensive alternatives to biologics, biosimilars have served to increase access to care for patients with chronic or other health conditions in the MENA region [3,4]. Increasing disease rates, variable healthcare accessibility, and a growing older population has made the MENA a vital area for such drugs, where the demand for biologics outpaces the general affordability of such medicines, making such clinically similar alternatives prime targets for development and marketing [4,5]. As of 2020, biosimilars have already had over a decade of success in the MENA in treating chronic conditions, such as Crohn’s Disease, diabetes, and additional autoimmune conditions, using biosimilar comparators to infliximab, etanercept, insulin glargine, and adalimumab—the four leaders in the market that year [6]. Over the next decade, there is expected to be greater focus on biosimilar treatments for diabetes, autoimmune diseases, and oncology [6]. However, the regulatory frameworks in many African countries are still in the nascent stages, as many countries have yet to officially adopt any regulations for the approval of biosimilars [5,7]. This contrasts with others, such as Egypt, where over 55 biosimilars are on the market [8], as well as the Kingdom of Saudi Arabia (KSA) and Lebanon, having over 36 approvals [3] as of 2023. By 2013, Egypt (in North Africa) and Lebanon (in the Middle East) have explicitly utilized these recommendations in their official regulatory framework. Since that time, many other countries have made explicit references in their guidance or to the WHO as their international reference agency.

The Middle East and North Africa (MENA) region has, over the last few years, been deemed an influential emerging pharmaceutical market with biosimilars experiencing uptake in line with global market trends [7], with more countries expected to experience large-scale expansions in the coming decade [3]. Reports indicate that the MENA market had a USD 442.5 million value in 2020, with a projected increase to USD 623.7 million by 2027 [7]. In this region, the KSA was the market leader for biosimilars in 2023, followed by Egypt and the UAE [7]. Despite these successes, economic factors such as a heavy dependence on the current prices of oil cause many MENA countries to suffer turbulent economies, with impacts on spending capacity and healthcare expenditure [9]. Greater government flexibility and support would help provide areas for the expansion of this market, and the work by the WHO regarding biosimilar research and guidance has addressed these needs. 

In 2022, the WHO released the revised Guidelines on Evaluation of Biosimilars [10], henceforth referred to as the “2022 revision”, in order to provide a global standard for biosimilar licensing reflective of the latest advances in science and knowledge acquired from research and surveying the needs of member states [11]. The results are improvements to standardization, scope, and study procedures. Included are clarifications of terminologies, a reduction of redundant non-clinical studies, a minimization of animal testing, simplified clinical safety and efficacy requirements, and acceptance of the use of reference products not of local authorization as comparators [11,12]. The particular addition regarding reference products comes partly as a response to the real-world practice over the last decade of regulatory authorities to use such non-local or externally sourced reference products in comparability studies out of necessity and against recommendation, according to the International Generic and Biosimilar Medicines Association [12]. Additionally, there is the rationale that other jurisdictions have similar scientific and regulatory standards, and the local regulatory authority can request additional data supporting the acceptability of the non-local product [10].

The key attribute of a biosimilar is the similarity of the agent in terms of safety, quality, and efficacy to a licensed comparator product [10]. The WHO defines a “reference product” (RP) as the biological used as the comparator to establish such similarity through direct head-to-head comparability exercises to “establish similarity in quality, safety and efficacy” [10]. It is stressed within the WHO guidelines that this RP should be an originator product, as well as a biological of suitable quality. It should also feature market longevity, having already been licensed via a full registration dossier. Having a high-quality and consistent supply of the RP is a necessity for the approval of a new biosimilar in any jurisdiction.

The choice and sourcing of this RP can become an issue when the RP is difficult to procure, acquire in consistently manufactured batches, or expensive beyond the limits of drug trials [13]. These issues are exacerbated by the need for a rapid approval due to shifting market temperaments and expiring patent statuses of target pharmaceuticals. Sourcing may be performed directly by the manufacturer, which, in the interest of remaining competitively discreet, may not be viable. Alternative to or in conjunction with the activity of open-market sourcing is a second option to obtain the RP. Open market sourcing, however, has issues, including the potential to introduce batch-to-batch variability, impose local shortages, reduced study sustainability, and added time and expense of importation [14].

Yet, it is not always the case that a viable domestically licensed product exists to serve as an RP for the biosimilar approval process to succeed. Most countries have stipulations in guidelines pertaining to such externally sourced biologics for comparability tests. Many MENA countries are willing to source from the countries abiding closely to the FDA or EMA standards, such as the US or EU, but bridging studies will, in some cases, be required by the regulating authority to demonstrate a locally acceptable quality of product [15,16]. This is despite the fact that bridging studies are considered by some to be an “unnecessary burden”, generating additional expenses, while only serving to replicate existing study data [17]. This could create an environment in which neighboring jurisdictions are unable to cooperate effectively and either remain dependent on external suppliers or forgo standards of non-local sourcing. The release of the 2022 revision to the biosimilar guidance by the WHO addresses exactly this. However, the impact of this regulation has yet to be studied among the MENA countries.

The goal of this review was to compare the WHO 2022 revision to the original 2009 Guidelines on Evaluation of Similar Biotherapeutic Products (SBPs) salient to non-local reference products utilized in comparability studies by performing a literature review to identify MENA WHO member states either drafting or having biosimilar guidance which accepts or adopts WHO recommendations. This review aims to examine the developing impact of this 2022 revision on the guidance of MENA countries for non-local comparator sourcing in order to inform the overall evaluation of the speed of uptake and relative desire to harmonize in this region, where, historically, there has been an absence of regional consensus. While there are many additional potential factors to explain the variance in uptake between the different MENA countries, these fall outside the scope of this review.

## 2. Materials and Methods

The countries in this review were selected based on WHO membership and being a member of the MENA, as defined by the World Bank [18], and have either adopted, referenced, or are currently drafting biosimilar-specific regulation explicitly adherent to any WHO guideline (2009 guidelines or 2022 revision). For any country, the latter criterion specifically required a statement within the official biosimilars guidance or as determined in the 2020 IQVIA whitepaper on the matter [19].

The main evidence comprising the results for this study was exclusively obtained from the national health institutions of the countries included by review of the official regulatory documents pertaining to the evaluation of biosimilars for each of the selected nations by country-level regulatory agency website. Guidance documents were located via comprehensive, domain-specific searches in internationally available search engines, using the term “biosimilar”, within the official website of each regulating authority by nation. Official domain queries were performed first in English and then secondly in the official language of the country if no results were returned. It was preferable to use only English versions of guidelines to avoid potential translation issues. Care was also taken to query relevant country-specific biosimilar terminologies in cases where the local national regulatory authority (NRA) still used alternative terms in place of “biosimilar”. This method was used systematically to reduce bias and maximize inclusion of the possible relevant materials, encompassing not just guidance but replacements and revisions as well. 

The current state of the MENA guidance on the sourcing of non-local reference products was characterized. In particular, the detailed stance is described, along with terminology, licensing, dosage, and route of administration (ROA), compared to the reference product. Country-level guidance on sourcing non-local products was detailed and compared, with specific focus on features related to accessibility. This analysis included delineating to the extent possible which regions are acceptable to source from, source characteristics, requirement of bridging studies between local and non-local reference products, and specific criteria relevant to the jurisdiction. The responsiveness of member states to the WHO revision was assessed, as well as the evaluation of the effectiveness of WHO recommendations.

## 3. Results

Several important changes were apparent in the 2022 revision (Table 1). The phrases “similar biotherapeutic product” and “reference biotherapeutic product” were replaced with “biosimilar” and “reference product”, respectively. Slight modifications to the language of licensing, dosage, and the ROA as biosimilar and RP are compared to, in part, allow for flexibility in the main change of the acceptance of non-local comparators as RPs. While the 2022 revision still requires that RPs be widely marketed products in a licensed jurisdiction, general acceptance is afforded for non-locally sourced comparators. This contrasts with the original guidance, where it was the responsibility of the NRA to establish country-specific criteria for acceptable jurisdictions. 

Of the 19 MENA countries, it was determined that Egypt, Lebanon, Morocco, Tunisia, and the UAE met the search inclusion criteria in that those countries were either drafting or had published guidance specific to biosimilars with reference to the WHO recommendations. The key guidelines for non-local RP policy among these countries are provided in Table 2. Only Egypt [20], Lebanon [21], Morocco [22], and Tunisia [23] have currently published guidance publicly accessible on the domain of the respective NRA for that country. Morocco and the UAE have no completed guidance available publicly on the official NRA website specifically pertaining to biosimilars since 2020 [6,24] or at the time of writing this paper. However, these countries do accept WHO as an international reference or as the international reference body [7], and many follow established policy and guidance for the registration of biosimilars, as well as the use of local or imported drugs without country-specific guidelines in place, such as the UAE [24]. 

## 4. Discussion

Biosimilar accessibility and adoption has demonstrated variable success [7] in the MENA to date. Promising and successful adoption is seen in a few countries, such as Egypt, KSA, UAE, and Algeria, while adoption is slower in countries like Morocco, partially due to the time and expense in sourcing RPs for comparability studies from non-local sources [25]. The 2022 revision by the WHO of biosimilar evaluation guidelines appeared to address this issue by legitimizing an existing norm of sourcing from local jurisdictions. This recommendation comes alongside many other major alleviations built upon the last decade of study and research in biosimilars which agree that the previous precedent of non-clinical and bridging studies were likely an unnecessary hindrance in many cases [11,12,15,16]. These recommendations had cost-saving potential by reducing studies and expediting approval, which, particularly in the MENA, is a key interest in policy development. A successful future for biosimilars in the MENA region therefore hinges on the adaptability to the 2022 revision and similar initiatives by the EMA and FDA [3].

Compared to the language of the original 2009 guidelines, the 2022 revision provides a much more flexible and open-ended NRA-centric framework for biosimilar approval, which, in its generality, aims to anticipate the heterogeneity of countries within its scope, in addition to the differences within biosimilars covered. The WHO maintains the need for local NRAs to determine the threshold of evidence required to use non-locally sourced RPs across both versions, but the 2022 version explicitly accepts other jurisdictions having “similar scientific and regulatory standards”. Language regarding jurisdiction remains the same, requiring that RPs be from jurisdictions experienced with evaluating biotherapeutic or biological products having “well-established regulatory frameworks”, along with defined and effective post-market surveillance strategies. The 2022 revision expand on the stated acceptability criteria for non-local RPs by adding that, should bridging studies be necessary, additional PK studies be implemented. Lastly, the 2022 revision affords some leniency to accommodate differences in RP sourcing locales by endorsing the usage of RPs having a different “pharmaceutical form, formulation, excipients, and presentation” than the biosimilar, given sufficient justification and that the dosage and ROA remain the same.

While the 2022 revision has been released for over a year, there has been only a limited perceptible effect observed in the guidance of the MENA countries. Of the MENA countries included in this review, only Egypt released a revised biosimilars guidance in 2023 with reference to the WHO. However, previous Egyptian guidelines from 2020 still allowed referenced products sourced from marketed authorities such as the US or EU. Hence, practically speaking, this does not serve as a major change in this aspect for Egypt. Lebanon and Tunisia both have guidelines that reflect the prior 2009 WHO recommendations, with Lebanon still using the former term “Similar Biotherapeutic Product” in its official document. Despite the need for these less expensive biologics, developing North African countries, including Morocco and Tunisia, have historically been slow to establish national legal or regulatory frameworks in part due to a lack of consensus on policy due to differences in healthcare systems [26].

It is a logical justification that biologicals approved in jurisdictions having the same standards need not be revalidated locally with additional studies. In some ways, this revision is a welcome streamlining of a previously convoluted framework. However, as countries like Morocco continue to demonstrate slow adoption, it may appear that the WHO guidelines are too permissive and, therefore, unattractive to implement when compared to those of the US FDA and EMA. These two major authorities have more stringent approval criteria, which many other MENA countries have adopted instead to relative success (KSA, UAE, Kuwait, and Algeria, for example). Indeed, in the 2015 Jordan Food and Drug Administration Principles for Registering Similar Biological Medicines [27], this sentiment is echoed, describing the EMA as having “the most well developed regulatory framework for biosimilars”. 

There remains a vast unmet need for inexpensive and effective biologics in parts of the MENA, and the 2022 revision appears to have had a modest uptake in the effort to harmonize regulation and cooperation in the region [28,29,30,31]. In light of these developments, the current state of this observed disharmony of outcomes is likely a reflection of the multidimensional heterogeneity of the healthcare systems, infrastructure and needs, economics, and regulatory capacities and maturity among the counties in this region. The impact in these areas will be an important point of study in reference to the 2022 revision. A comparison of outcomes in countries such as Egypt, abiding by the new revision, and those such as the KSA, which has found success using the guidance by the EMA, is warranted. Whether the more flexible recommendations on non-local sourcing will manifest tangible benefits in studies is still to be determined. Perhaps more critically, additional research is needed to ascertain whether there are drawbacks associated with overly variable regulatory standards between jurisdictions, as well as substandard or questionable products harming the quality of data generated in studies using flexible sourcing. It is agreed that the public health needs of the coming decade necessitate more MENA countries adopting the policies of the FDA, EMA, and WHO to streamline the approval process [3], and the latest update by the WHO is a critical step towards harmonization and increased availability of effective medicines in the emerging and developing markets of the MENA.

## 5. Limitations

The scope of this analysis was affected by the availability of completed guidance documents from Morocco and the UAE for this evaluation, with the latter particularly due to the prominence of the UAE in the Middle Eastern biologics market. The lack of an internal insight into the progress of the NRAs in drafting guidance further compounds this issue, as this review relies on publicly available documents for which there may not yet be any preliminary announcement or release. Additionally, the follow-up time to the latest WHO revision in November of 2022 may not have been extensive enough for all applicable countries to have revised guidance. It will be important to re-examine the adoption rates once more time for revision has been allotted, particularly when all countries have officially drafted guidelines available for comparison.

## 6. Conclusions

The 2022 revision and the recommendation to source RPs for non-local jurisdictions have had a limited uptake after more than a year of release. Official adoption was limited heretofore to the Egyptian Drug Authority. It appears that the majority of MENA countries continue to rely on the 2009 guidelines or EMA recommendations. Given that additional flexibility is afforded by the 2022 revision compared to the 2009 guidelines, it is likely that adoption rates will accelerate as the need for biosimilars intensifies in the coming decade. Our future planned studies include evaluation of the evolving impact of these recommendations in the MENA.

## Figures and Tables

**Table 1:** Comparison of original WHO guidance (2009) and the revision (2022).

	2009—Guidelines on Evaluation of Similar Biotherapeutic Products (SBOs), Annex 2, TRS No. 977	2022—Guidelines on Evaluation of Biosimilars TRS 1043, Annex 3
Terminology		
Similar biologic	Similar biotherapeutic product	Biosimilar
Comparator	Reference biotherapeutic product	Reference product
Non-local RP sourcing		
Stance on non-local jurisdiction RP sourcing	The WHO recommends a nationally licensed RP, but an NRA may establish additional criteria to use RPs sourced from other countries. It is further recommended to consider economic factors such as market experience, duration, and volume.	It is possible to use an RP sourced from another jurisdiction. NRA will determine if additional supporting information is required to approve the non-local product for use in comparability studies.
Jurisdiction of RP	The RP should be licensed and widely marketed in a jurisdiction having a well-established regulatory framework, principles, and experience evaluating biotherapeutic products and post-market surveillance.	The RP should be from a licensed jurisdiction with a well-established regulatory framework and experience evaluating biotherapeutic products and post-market surveillance.
Requirements for bridging studies	NRAs are recommended to establish criteria of acceptability for RPs.	If required, bridging studies between local and non-local RPs should be stringent and may require additional PK bridging studies.
Other considerations		
Usage of RP in comparator studies	The RP must be the same throughout development.	The RP can only be a single biological product from a single authorizer which may be from another jurisdiction.
Dosage and ROA	The biosimilar must have same dosage and ROA as RP.	The biosimilar must have same dosage and ROA as RP, but the strength, pharmaceutical form, formulation, excipients, and presentation (device) may be different than the RP with appropriate justification.
Additional criteria	The use of a non-local RP in comparability exercises does not imply approval in the local country.	NRAs must determine sufficient information supporting safe and efficacious use required for a non-local RP.

WHO, World Health Organization; RP, reference product; ROA, route of administration; NRA, national regulatory authority.

**Table 2:** Latest MENA WHO member-state biosimilar guidance status following WHO 2022 guideline revision as it relates to sourcing non-local comparators.

Country	Egypt	Lebanon	Morocco	Tunisia	UAE
Guidance sourcing					
NRA	EDA	MOPH	DMP	DMP	MOHAP
Guidance accessible	Yes	Yes	Partial	Yes	No
Terminology for biosimilars	Biosimilar	Similar biotherapeutic product	Biosimilar	Biosimilar (Similar biological medicine)	Biosimilar
Adopted WHO 2022 revision	Yes	No	No	No	NA
Non-local RPs					
Non-local RP sourcing	Products registered outside of Egypt may be used if the comparator is from a “stringent authority” (EU or US for example) and is “representative of the reference medicinal product”. The applicant will qualify to import enough for comparability studies. Non-local RPs are not considered approved in Egypt.	May use externally sourced RPs given sufficient justification according to WHO 2009 standards.	NS	In general, the RP should be registered in Tunisa. However, a few regions with market authorization are acceptable, given a signature from the Central Pharmacy of Tunisa, as external sources: EU, UK, US, Australia, Canada, and/or Japan.	NA
Usage of RP in comparator studies	The RP must be the same throughout development.	The RP must be the same throughout development.	NS	The same RP needs to be used throughout comparability exercises.	NA
Additional RP sourcing specifications	Multiple reference lots of RP are recommended to control for batch-to-batch variation. Random sampling of lots is desired. Sourcing should be performed over an extended period, if possible. Should be the same batches as used in clinical comparison studies. RPs should be stored and transported under recommended conditions within their shelf lives.	No additional sourcing requirements are explicitly specified. The 2009 guidance by the WHO informs sourcing policy.	NS	Reference products not registered in Tunisia or available on the Tunisian market must be approved first for importation. A request must be made to the DPM before authorization from the Central Pharmacy of Tunisia (PCT) may be granted.	NA
Dosage and ROA	RP should be the same dose and ROA as biosimilar.	The biosimilar must have the same dosage and ROA as RP.	NS	The biosimilar should be the same pharmaceutical form, dosage, and ROA as the RP.	NA

EDA, Egyptian Drug Authority; MOPH, Ministry of Public Health; DMP, Directorate of Medicine and Pharmacy/Direction de la Pharmacie et du Medicament; MOHAP, Ministry of Health and Prevention; RP, reference product; ROA, route of administration; NA, not available; NS, not specified; WHO, World Health Organization; EU, European Union; US, United States; UK, United Kingdom.

## Data Availability

All data utilized for this review is publicly available and listed in References of this manuscript.
